# MicroRNA Methylome Signature and Their Functional Roles in Colorectal Cancer Diagnosis, Prognosis, and Chemoresistance

**DOI:** 10.3390/ijms23137281

**Published:** 2022-06-30

**Authors:** Rashidah Baharudin, Nurul Qistina Rus Bakarurraini, Imilia Ismail, Learn-Han Lee, Nurul Syakima Ab Mutalib

**Affiliations:** 1 UKM Medical Molecular Biology Institute (UMBI), Universiti Kebangsaan Malaysia, Kuala Lumpur 56000, Wilayah Persekutuan Kuala Lumpur, Malaysia; ieda_baharudin@yahoo.com; 2 Faculty of Applied Sciences, Universiti Teknologi Mara (UiTM), Shah Alam 40450, Selangor, Malaysia; nurulqistina2312@gmail.com; 3 Faculty of Health Sciences, School of Biomedicine, Universiti Sultan Zainal Abidin (UniSZA), Kuala Nerus 21300, Terengganu, Malaysia; imilia@unisza.edu.my; 4 Novel Bacteria and Drug Discovery Research Group, Microbiome and Bioresource Research Strength, Jeffrey Cheah School of Medicine and Health Sciences, Monash University Malaysia, Subang Jaya 47500, Selangor, Malaysia; 5 Faculty of Health Sciences, Universiti Kebangsaan Malaysia, Kuala Lumpur 50300, Wilayah Persekutuan Kuala Lumpur, Malaysia

**Keywords:** microRNA, colorectal cancer, epigenetics, methylation, biomarker

## Abstract

Colorectal cancer (CRC) is one of the leading causes of cancer-related deaths worldwide. Despite significant advances in the diagnostic services and patient care, several gaps remain to be addressed, from early detection, to identifying prognostic variables, effective treatment for the metastatic disease, and the implementation of tailored treatment strategies. MicroRNAs, the short non-coding RNA species, are deregulated in CRC and play a significant role in the occurrence and progression. Nevertheless, microRNA research has historically been based on expression levels to determine its biological significance. The exact mechanism underpinning microRNA deregulation in cancer has yet to be elucidated, but several studies have demonstrated that epigenetic mechanisms play important roles in the regulation of microRNA expression, particularly DNA methylation. However, the methylation profiles of microRNAs remain unknown in CRC patients. Methylation is the next major paradigm shift in cancer detection since large-scale epigenetic alterations are potentially better in identifying and classifying cancers at an earlier stage than somatic mutations. This review aims to provide insight into the current state of understanding of microRNA methylation in CRC. The new knowledge from this study can be utilized for personalized health diagnostics, disease prediction, and monitoring of treatment.

## 1. Introduction

Colorectal cancer (CRC) is one of the most commonly diagnosed cancers worldwide. The economic burden of CRC management of new cases in Malaysia is estimated at MYR 62 million per year [1]. While there have been significant advancements in diagnostic services and patient care, several gaps remain, from early detection to the identification of prognostic variables, the effective treatment of metastatic disease, and the implementation of customized treatment strategies. A quite recent study concludes that CRC is an expensive disease, with provider costs ranging from MYR 13,672 for stage I to MYR 27,972 for stage IV [2].

Cancer is a global burden with over 14.1 million new incidences in 2012 and is projected to increase in the next decade. In Malaysia, CRC is the most common cancer, with an overall incidence rate of 21.3 cases per 100,000 population [3]. Many CRC studies have revealed molecular alterations involved in its pathogenesis [4,5,6], yet the prognosis of advanced CRC is still dismal and the search continues for biomarkers which could accurately guide the medical practitioners in the management and treatment of CRC. Therefore, robust prognostic and predictive biomarkers are undoubtedly an important goal. One of the candidates for the biomarkers could be discovered by analyzing the epigenome of the tumors.

MicroRNAs are small (~22 nucleotides), non-coding RNAs that modulate gene expression in various eukaryotes [7]. These single-stranded RNAs exert their roles by interacting with specific target mRNAs through partial complementarity with sequences located mainly in the 3′UTR, subsequently causing mRNA degradation or translational inhibition [7]. MicroRNAs perform critical roles in a variety of cellular processes, including apoptosis, cell cycle, proliferation, differentiation, and angiogenesis, by simultaneously regulating the expression levels of several genes. MicroRNAs are found in all tissues and play a function in every cell type [7]. A large number of studies in CRC have also revealed that microRNA expression profiles change remarkably between normal tissues and tumors, were associated with drug resistance, as well as possess diagnostic, prognostic, and theranostic values [8,9,10,11]. Moreover, microRNAs play dual roles as oncogenes and tumor suppressors, which is the key function in tumorigenesis [12].

**Figure 1:**
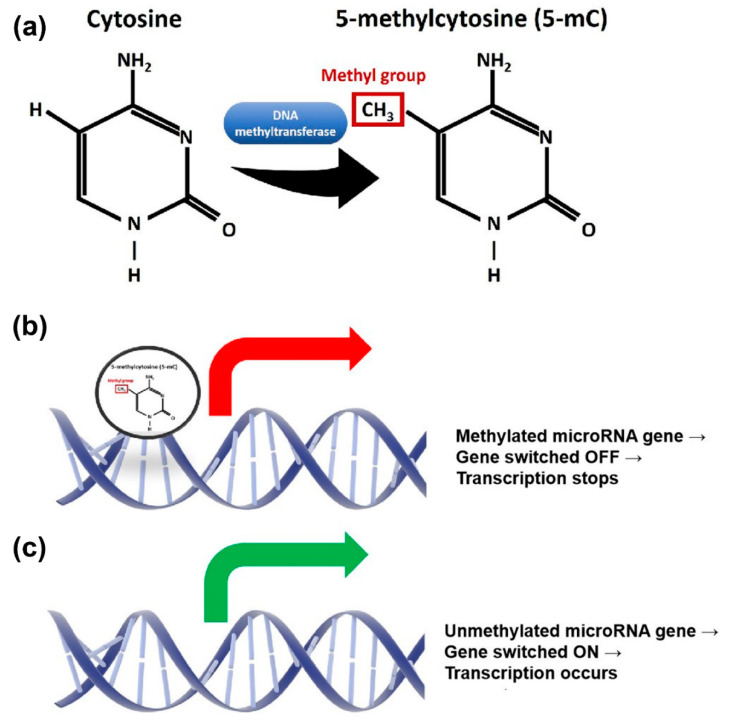
Overview of the methylation process. (**a**) Methylation is defined as an addition of a methyl group to the cytosine ring. (**b**) When the microRNA gene is methylated, it will be switched off and the transcription process will stop. No mature microRNA will be expressed. (**c**) When there is absence of methylation, the microRNA gene will be switched on, therefore transcription will occur and mature microRNA will be expressed.

Although the specific mechanism underlying microRNA deregulation in cancer has yet to be determined, multiple studies have demonstrated that epigenetic mechanisms play a significant role in the regulation of microRNA expression in cancer cells [13,14,15], particularly DNA methylation, which is a biological process that adds methyl groups (CH_3_) to the cytosine ring, thus producing 5-methylcytosine (5mC) (Figure 1a). Expression of microRNAs might be epigenetically regulated via DNA methylation of CpG islands located at promoter regions [13] (Figure 1b,c). Alterations in those mechanisms might perturb microRNAs expression, subsequently altering gene and protein expression, leading to cancer progression. The study of DNA methylation in microRNA genes is not entirely new. Publications on this topic began to emerge around a decade ago, yet the gap in knowledge remains, particularly in CRC. Most of the published data have been derived from CRC cell lines and not from clinical specimens [16,17]. In addition, to the best of our knowledge, there has been a limited number of publications on genome-wide microRNA methylome profiling in this cancer [18]. Most of the studies were focused on the selected microRNAs known to be hypermethylated in CRC, such as miR-34b/c, miR-124, miR-133b, and miR-324, etc. [19,20,21]. While reports on epigenome-wide microRNA methylation profiles have already been published in other cancers, such as pancreatic, breast, and oral cancers [22,23,24], studies on CRC are severely lacking.

## 2. MicroRNA Methylation with Diagnostic and Prognostic Markers

MicroRNAs may function as tumor suppressor genes, and their downregulation is commonly detected in CRCs. Epigenetic regulation, including DNA methylation, is one of the mechanisms associated with microRNA silencing. Since epigenetic silencing is a reversible process, aberrant methylation of microRNA emerges as a novel class of biomarkers, with strong potential as diagnostic and prognostic markers in CRCs.

### 2.1. MiRNA-124a

MiR-124a is among the first microRNAs in CRC that have been shown to be silenced via an epigenetic mechanism [17,25]. It was discovered through a genetic unmasking experiment from a cell line model with disrupted DNA methyltransferases [17]. DNA methylation of miR-124a may serve as an epigenetic biomarker for CRC, since this gene is more frequently methylated in CRCs than in other cancers [26]. Lujambio et al. have demonstrated that the epigenetic silencing of miR-124a via CpG island hypermethylation leads to the activation of cyclin D kinase 6 (CDK6), an oncogene, and the phosphorylation of the retinoblastoma (Rb) tumor suppressor gene [17]. The authors proved that miR-124a was specifically methylated in cancer cells, suggesting a tumor suppressive role [17]. Encoded by three independent loci (miR-124a-1, -2, and -3), miR-124a is associated with various CpG islands [13]. Aberrant DNA methylation of miR-124a-1, -2, and -3 was detected in bowel lavage fluid (BLF) specimen in CRC patients. Among these three genes, methylated miR-124a-3 showed the greatest sensitivity for CRC detection, highlighting the potential of this microRNA as a non-invasive diagnostic marker for CRC screening [27].

Ueda et al. [28] also reported that three miR-124a genes were methylated during carcinogenesis in patients with ulcerative colitis (UC). Nonetheless, methylation of miR-124a-3 was frequently detected in the early stage of colitis-associated cancer (CAC), indicating the importance of this microRNA in estimating the individual risk of developing CAC. Moreover, another related study found that miR-124a was methylated in UC patients with CRC. The authors identified higher methylation level in rectal tissues in an age-dependent manner [29]. Considered together, these two studies suggest that methylation of miR-124a is a potential marker for identifying UC patients with high risk of developing CRC.

Numerous studies have demonstrated the role of miR-124a as a prognostic biomarker in CRC patients. The miR-124a expression level varied depending on the tumor differentiation grades, while the low miR-124a was measured in tissues with moderate to poor differentiation. In addition, survival analysis of 96 CRC patients showed that the group with downregulated miR-124a exhibited worse prognosis in overall survival (OS) and disease-free survival (DFS) [30]. Similarly, Jinushi et al. [31] discovered the low expression of miR-124a in plasma samples of CRC patients with poor OS. Moreover, another study found that downregulation of miR-124a could induce cell proliferation, migration, invasion, and metastasis in CRC by negatively regulated ROCK1 expression [32]. In the future, miR-124a may constitute an effective new prognostic biomarker for CRC patients with advanced disease or metastasis.

### 2.2. MiRNA-137

MiR-137 is located on the chromosome 1p22 within the gene sequence of MIR137HG [33]. This microRNA is embedded in a CpG island and is often downregulated in several tumors, including CRC, due to the promoter hypermethylation [34,35,36]. Several studies have reported the potential use of miR-137 methylation as a diagnostic biomarker. In a study by Balaguer et al., miR-137 was epigenetically silenced in CRC cell lines. In addition, the authors investigated the methylation status of miR-137 in CRC tissues and its adjacent normal. They discovered that the methylation of miR-137 is tumor-specific, considering that higher methylation was significantly detected in CRCs compared with normal tissues. Interestingly, a similar methylation frequency of miR-137 was observed in CRCs and adenomas, indicating that the methylation of miR-137 may occur in the early event of colorectal carcinogenesis [35]. This finding is in agreement with Kashani et al. [36], in which methylation of miR-137 occurred in CRCs and no methylation was observed in normal tissues. In addition, this study revealed increased hypermethylation of miR-137 in patients with a family history of CRC or other gastrointestinal-related cancers. These encapsulate the crucial role of methylation in miR-137 as a diagnostic biomarker in CRC.

CRC arises from abnormal growth of colon epithelium and subsequently transforms to adenomatous polyps, which over time progress to cancer. A study by Huang et al. observed a gradual decrease in miR-137 expression during the process of colorectal carcinogenesis. Therefore, they postulated that DNA methylation subsequently downregulates miR-137 in polyps is an early event in the development of CRC [34]. As discussed earlier, methylation of miR-124 could be a valuable marker in identifying UC patients with high risk of developing CRC. In addition, the authors discovered the potential of methylation in miR-137 as an independent risk factor in differentiating UC patients with high risk of developing CRC. Moreover, methylation of this microRNA showed a substantial AUC value in discriminating UC patients with high or low risk of developing cancer, further demonstrating its importance in CRC screening [29].

Dysregulation of miR-137 is associated with prognosis of CRC. The decline of miR-137 expression is able to predict recurrence and survival of stage II CRC patients [37]. Furthermore, altered miR-137 expression has been shown to be associated with the progression of CRC. Through an in vitro model, downregulation of this microRNA induces cell proliferation, migration, and invasion in CRC by hindering the expression of TCF4. However, miR-137 could also target other downstream genes in addition to TCF4 to promote tumor progression. A study by Sakaguchi et al. demonstrated the capability of ectopic expression of miR-137 to suppress the tumorigenicity of colon cancer stem cells without affecting normal cells. Furthermore, they discovered that the presence of miR-137 restrained the colon cancer metastasis through the downregulation of DCLK1 expression [38]. Interestingly, research by Chen et al. suggests that miR-137 expression in CRC is subject to epigenetic silencing mediated by Mecp2, a DNA methyl CpG binding protein. Mecp2 can directly bind to the promoter region of miR-137 and lead to a decrease in expression. Restoring the expression of miR-137 led to the inhibition of the colorectal tumor growth in a xenograft model, as well as in vivo hepatic metastasis [39].

### 2.3. MiRNA-34

MiR-34, a tumor suppressive microRNA family, has been observed to be directly regulated by the tumor suppressor p53 [40]. The miR-34 family consists of three members, including miR-34a, miR-34b, and miR-34c. Interestingly, three miR-34 family members are produced by two different transcriptional units [41]. Human miR-34a is located at chromosome 1p36.22, whereas miR-34b and miR-34c reside on chromosome 11q23.1 [41]. MiR-34 is frequently methylated in CRC tissues and to a lesser extent in adjacent normal tissues. Notably, Wu et al. [42] discovered that methylation of miR-34a was observed in 76.8% of CRCs and 5% of healthy volunteer stool samples. Intriguingly, miR-34b/c methylation was displayed in 93.6% of CRC stool samples and no methylation was observed in the healthy samples. This finding is consistent with those of Kalimutho et al., whereby they found that 75% of fecal CRC patients exhibited aberrant methylation of miR-34b/c [43]. High sensitivity detection of methylation miR-34b/c in stool samples may be an effective non-invasive screening method for the diagnosis of CRC.

MiR-34a expression is useful for CRC prognosis. Gao et al. evaluated the expression of miR-34a-5p in recurrence and non-recurrence groups of stage II and stage III CRC patients. Their results revealed that miR-34a-5p was downregulated in the recurrence group despite the TNM stage. In addition, the elevated expression of this microRNA was directly proportional to DFS. This suggests that miR-34a-5p is a potential prognostic marker to predict the aggressiveness of cancer in stage II and III CRCs. Moreover, the authors discovered that the inhibition of metastatic properties in CRCs is a p53-dependent manner [44]. The downregulation of miR-34a in CRC is presumably caused by the aberrant methylation at the promoter region. High methylation frequency of miR-34a has been observed in primary tumors that have developed liver and lymph node metastases. Furthermore, silencing of this microRNA was associated with an increased expression of c-Met and β-catenin, which exhibited pro-metastatic function. Therefore, the epigenetic silencing of miR-34a together with upregulation of c-Met and β-catenin in primary colon cancer may have a prognostic value to identify patients with a high risk of liver metastases [45].

However, two studies presented the opposite findings. Rapti et al. showed that miR-34a was overexpressed in poorly differentiated CRC, which is highest in grade III tumors as compared with the lower grades. Deregulation of this microRNA leads to worsened DFS and OS, independently of clinicopathological factors, such as tumor size, histological grade, tumor invasion, and nodal status apart from distant metastasis. Therefore, elevated miR-34a expression is a potential unfavorable prognosis marker in CRC [46]. Another study by Hasakova et al. found that miR-34a-5p was upregulated in CRCs as compared with the adjacent tissues. Nevertheless, they found that the expression of miR-34a-5p varied in accordance with the sex, whereby downregulation of this microRNA was ascertained in male patients rather than females. In addition, a better survival rate was observed in male patients who exhibited high miR-34a-5p, and was unlikely associated with advanced stages [47]. A possible explanation of these contrasting results is the tumor microenvironment heterogeneity of CRCs.

### 2.4. Other microRNA Genes

MiR-133b is a tumor suppressor gene and is often silenced in CRC [48]. Silencing of this microRNA is correlated with CpG methylation in the promoter region. In addition, miR-133b was reported to be downregulated in the primary CRC and metastatic hepatic tissues. Remarkably, miR-133b negatively regulates the HOXA9/ZEB1 pathway, which then promotes tumor metastases and poor outcomes in CRCs [49]. DNA hypermethylation of miR-1 was first observed in hepatocellular carcinoma (HCC) primary tissues and cells [50]. Later, Chen et al. discovered the methylation of miR-1 in primary CRC tissues [51]. In addition, the DNA methylation-mediated downregulation of miR-1 was observed in 12 out of 14 colon cancer metastases. Interestingly, miR-1 was shown to interact with miR-133a in CRC and concurrent silencing of these microRNAs negatively regulate TAGLN2 expression. Therefore, miR-1-133a interaction with upregulation of TAGLN2 has a significant role in CRC metastasis.

The expression of miR-9 may be regulated by DNA methylation and histone modification in CRC. Methylation of this microRNA was detected in 56% of primary CRC. However, high methylation frequency was observed in advanced stages of CRC with regional nodal and vascular invasion aside from metastasis. The finding of this study showed that miR-9 silencing is crucially involved in CRC progression [52]. Moreover, deregulation of miR-9 has been reported to promote proliferation and tumor cell survival in CRC [53].

In addition to the microRNAs mentioned above, miR-345 and miR-342 are highly methylated, with low expression in CRCs in comparison with non-cancerous tissues [54,55]. Ectopic expression of these microRNAs is able to suppress colon cancer proliferation and invasiveness. Tang et al. discovered that miR-345 inhibits tumor growth by targeting BCL2-associated athanogene 3 (BAG3), a molecule that regulates the apoptosis process [54]. In contrast, restoration of miR-342 has been found to reduce the expression of DNMT1, which subsequently demethylates tumor suppressor genes, such as ADAM23, HINT1, RASSF1A, and RECK in CRC [55].

**Figure 2:**
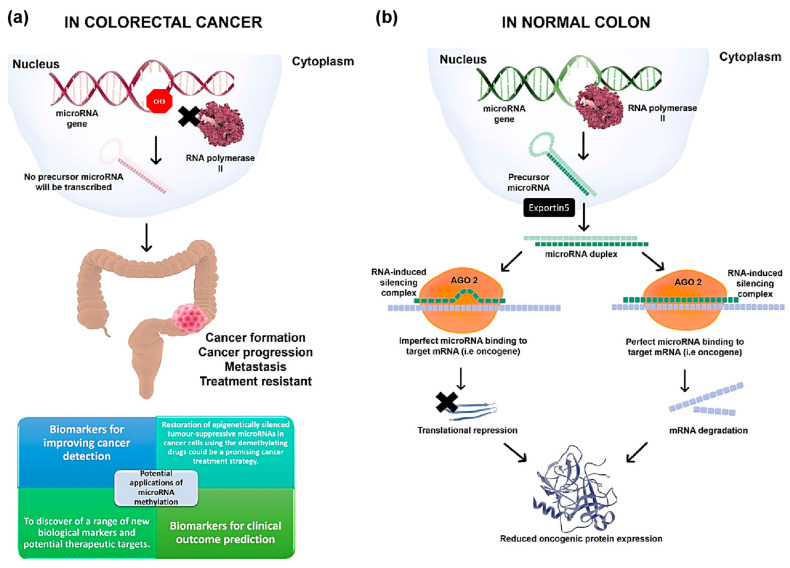
The involvement of methylated microRNA in CRC. (**a**) Simplified illustration of microRNA and its potential involvement in CRC. DNA methylation is a key epigenetic mechanism for silencing RNA polymerase II-transcribed genes [71]. When the microRNA gene is methylated, no precursor microRNA will be transcribed, thus reducing its mature microRNA expression [72]. This in turn could lead to cancer formation, progression, and treatment resistance. (**b**) Simplified illustration of microRNA and its potential involvement and application in normal colon. The unmethylated microRNA gene will lead to transcription of microRNA precursor by RNA polymerase II, which will then be exported into the cytoplasm by Exportin 5, followed by processing with the RISC, which will result in target gene translation repression or mRNA degradation. As a result, oncogenic protein expression will be reduced.

A non-exhaustive compiled summary of microRNA methylation implicated in CRC is presented in Table 1. Clearly, epigenome-wide profiling of microRNA methylation using high-throughput approaches, such as microarray or whole-genome bisulfide sequencing has not been performed, further highlighting the importance of our study. Finally, an illustration on the involvement of microRNA methylation in CRC progression is provided in Figure 2a,b.

**Table 1:** Snapshot of methylation-sensitive microRNAs implicated in CRC.

MicroRNA(s) and Reference	MicroRNA Methylation Detection Method	Types of Specimens	Key Findings
miR-124a [17] Known targets: STAT3, IASPP PRRX1, KITENIN, PRPS1, RPIA PTB1/PKM1/PKM2, DNMT3B, DNMT1, ROCK1, PRRX1, PLCB1	Methylation-specific PCR (MSP) and bisulfite sequencing	Cell line model with disrupted DNA methyltransferase	Epigenetic silencing of miR-124a via CpG island hypermethylation leads to CDK6 oncogene activation and Rb phosphorylation
miR-34b/c [19] Known targets: SATB2	Methylation-specific PCR (MSP) and bisulfite sequencing	CRC cell lines	miR-34b/c and NTG4 are novel tumor suppressors in CRC miR-34b/c CpG island is a frequent target of epigenetic silencing in CRC
miR-133b [20] Known targets: CXCR4, HOXA9	Methylation-specific PCR (MSP) and combined bisulfite restriction analysis (COBRA)	Screening using CRC cell lines and validation in the tissues (6 CRCs, 2 adjacent non-tumors, and 2 healthy colorectal tissues)	miR-133b promoter hypermethylation is upregulated in CRC tissues The regulation of miR-133b methylation has potential therapeutic utility for CRC treatment
miR-324 [21] Known targets: ELAVL1	Methylation-specific PCR (MSP) and bisulfite sequencing	42 CRCs, 9 colorectal adenomas, and 16 normal mucosae in patients with and without CRC	Methylation at the EVL/miR-342 locus was identified in 86% CRCs and in 67% adenomas, suggesting that it is an early event in CRC carcinogenesis
miR-137, miR- 342 [36] Known targets: miR-137: TCF4, FMNL2, Aurora-A miR-342: DNMT1, FOXM1, FOXQ1	Methylation-specific PCR (MSP)	Fresh-frozen tissues (51 polyps, 8 tumors, and 14 normal mucosa)	miR-137 hypermethylation is higher in male patients miR-342 hypermethylation is associated with patients’ age
miR-9, miR-129, miR-137 [52] Known targets: miR-9: TM4SF1, FOXP2, ANO1 miR-129: MALAT1 miR-137: TCF4, FMNL2, Aurora-A	Methylation-specific PCR (MSP) and bisulfite sequencing	CRC cell lines and 50 primary CRCs with adjacent normal tissues	miR-9-1, miR-129-2, and miR-137 methylation occurred commonly in CRC cell lines and primary CRC tumors, but not in normal colonic mucosa miR-9-1 methylation was associated with lymph node metastasis
miR-345 [54] No known target	Methylation-specific PCR (MSP) and bisulfite sequencing	CRC cell lines and 31 CRC patients	miR-345 hypermethylation was detected in tumor vs. normal tissues and is associated with its low expression, lymph node metastasis, and worse histological type
miR-129-2, miR-345, miR-132 [56] Known targets: miR-129: MALAT1 miR-345: No known target miR-132: ZEB2, ERK1	Bisulfite sequencing and Methylation-Specific Multiplex Ligation-Dependent Probe Amplification (MS-MLPA)	CRC cell lines treated with 5-aza-2′deoxycytidine followed by validation in 205 CRCs	miR-345 and miR-132 hypermethylation is associated with a mismatch-repair deficiency in CRC miR-132 hypermethylation distinguished sporadic MMR-deficient CRC from Lynch-CRC
miR-132 [57] Known targets: miR-132: ZEB2, ERK1	Methylation-specific PCR (MSP) and bisulfite sequencing	CRC cell lines and 36 CRCs with adjacent normal tissues	miR-132 is epigenetically silenced in CRC cell lines and implies a poor prognosis in CRC
miR-1, miR-9, miR-124, miR-137 [29] Known targets: miR-1: SMAD3 miR-9: TM4SF1, FOXP2, ANO1 miR-124: STAT3, IASPP PRRX1, KITENIN, PRPS1, RPIA. PTB1/PKM1/PKM2, DNMT3B, DNMT1, ROCK1, PRRX1, PLCB1. miR-137: TCF4, FMNL2, Aurora-A	Quantitative bisulfite pyrosequencing	387 colorectal epithelial specimens (362 non-neoplastic and 25 neoplastic tissues)	Among patients with ulcerative colitis without neoplasia, the rectal tissues had significantly higher levels of microRNA methylation Methylation level was associated with age and duration of ulcerative colitis
miR-125 [58] Known targets: BCL2, BCL2L12, MCL1, SMURF1, VEGFA, TAZ, CXCL12/CXCR4	Bisulfite sequencing PCR	CRC tissues and adjacent normal tissues from 68 CRC patients	Patients with hypermethylation of miR-125a and miR-125b had a shorter life expectancy than those with normal levels
miR-941 [59] No known target	Bisulfite sequencing	CRC cell lines	Hypermethylated in HCT116 cells Suppresses cell growth and migration in CRC cells
miR-1237 [59] No known target	Bisulfite sequencing	CRC cell lines	Hypermethylated in HCT116 cells Transcriptionally independent from the host gene
miR-1247 [60] No known target	Methylation-specific PCR (MSP) and bisulfite sequencing	CRC cell lines and patients (hypermethylated and non-methylated CRCs)	Downregulated in methylated CRC and hypermethylated cell lines (RKO, HCT116) Novel tumor suppressor by targeting MYCBP2 in methylated CRC
miR-128 [61] Known targets: IRS1, Galectin-3	Bisulfite sequencing PCR	CRC cell lines and patients	miR-128 was epigenetically silenced by DNA methylation, implies a poor prognosis in CRC Restoration of miR-128 could inhibit cell proliferation by inducing cell cycle arrest
miR-148a [62] Known targets: BCL2, ERBB3	Bisulfite pyrosequencing	273 CRC patients (76 stage II, 125 stage III, 72 stage IV)	miR-148a was significantly downregulated in tumor stage III/IV and correlated with promoter hypermethylation Low miR-148a expression leads to poor therapeutic response and patients’ overall survival
miR-126 [63] Known targets: CXCR4	Methylation-specific PCR (MSP) and bisulfite sequencing	CRC cell lines and patients	Silencing of miR-126 in CRC tissue and cell lines was due to the promoter methylation Restoration of miR-126 inhibits VEGF expression, thus hindering tumor progression
miR-27b [64] Known targets: RAB3D	Methylation-specific PCR (MSP)	CRC cell lines	DNA hypermethylation of miR-27b CpG island decreases miR-27b expression Targets VEGFC to inhibit tumor growth and angiogenesis in vivo
miR-149 [65] Known targets: FOXM1, EPHB3	Methylation-specific PCR (MSP)	CRC cell lines	Treatment using polyphenol (BPIS) induces hypomethylation of miR-149 CpG island in HCT-8/Fu cells Upregulation of miR-149 improved chemosensitivity of CRC through miR-149/Akt-mediated cell cycle arrest
miR-497/195 [66] Known targets: IGF1R, NRDP1, KSR1, FRA-1, PTPN3, CARMA3, FGF2	Combined bisulfite restriction analysis (COBRA) and bisulfite genomic sequencing (BGS)	CRC cell lines and patients	Both miRNAs were hypermethylated and under expressed in precancerous lesion Pri-miR-497/195 was monoallelic methylated at CpG island in normal colorectal and biallelic methylated in most colorectal adenomas
miR-212 [67] Known targets: MnSOD	Methylation-specific PCR (MSP) and bisulfite sequencing	CRC cell lines and tissues	miR-212 was hypermethylated at upstream promoter region in CRC tissues and cell lines, but not in FHC cells Low miR-212 level associated with aggressive tumor phenotype and poor disease prognosis
miR-200c/141 [68] Known targets: ZEB1, DLC1, TRAF5	Methylation-specific PCR (MSP)	CRC tissues	miR-200c/141 cluster promoter region was significantly hypermethylated in colorectal tumors and adenomatous polyps, but not in hyperplastic polyp tissues
miR-373 [69] No known targets	Methylation-specific PCR (MSP) and bisulfite sequencing	CRC cell lines and 40 CRC patients	CpG island at promoter region of miR-373 was significantly hypermethylated in CRC tissues and cell lines May inhibit cell viability in CRC cell lines by targeting oncogene RAB22A

Known targets in CRC were identified using miRCancer database [70].

As previously mentioned, the methylome profiles of microRNAs in CRC patients have not been extensively characterized. While there are several published findings from our research group on DNA methylation profiles in CRC [6,73], none have focused on microRNA methylome in detail. MicroRNAs are considered the master regulators that control gene expression [74]. Therefore, research on the elements controlling microRNA is indispensable.

## 3. MicroRNA Methylation in CRC Chemoresistance

Emerging evidence has revealed that abnormal expression of microRNAs also plays a vital role in chemotherapeutic drug resistance. FOLFOX, which is a mixture of folic acid (FOL), 5-fluorouracil (F), and oxaliplatin (OX) [75], is one of the most extensively used chemotherapy regimens for the treatment of cancer, mainly CRC. While cancer treatment is progressing, the formation of chemoresistance clones have emerged as a significant obstacle in the clinic. Finding prospective biomarkers and therapeutic targets that could lead to an increase in the success rate of suggested therapies is critical to achieving a successful outcome. Since it has been established that microRNAs are significant participants in the biological system, researchers have become increasingly interested in understanding their functional activities. When it comes to overcoming chemoresistance to FOLFOX, microRNAs as post-transcriptional regulators have the potential to be extremely beneficial. A review on differentially expressed microRNAs involved in CRC chemoresistance was previously published by our group and should serve as complementary reading [76]. In this section, we will focus primarily on the methylated microRNAs and their roles in CRC chemoresistance.

MiR-26b expression was analyzed in 5-fluorouracil (5-FU) resistant CRC cell lines and parental cells. The results showed that miR-26b was significantly downregulated in the 5-FU resistant cell lines, and thus, it is probably involved in CRC chemoresistance. Importantly, the downregulation of miR-26b was associated with promoter methylation and treatment with a demethylating drug (5-aza-2′-deoxycytidine was able to restore the expression of miR-26b in resistant cell lines). Upregulation of miR-26b conferred 5-FU chemosensitivity by repressing PGP expression and further activating caspase-9 and caspase-3 [77].

Takahashi et al. have provided evidence that miR-148a is frequently downregulated through the promoter hypermethylation in the advanced CRC. Moreover, downregulation of miR-148a was significantly associated with a poor outcome in patients with stage III CRC treated with adjuvant 5-FU. In addition, low expression of this microRNA is associated with worse therapeutic response and survival rate in stage IV CRC patients treated with 5-FU and oxaliplatin chemotherapy [62].

Low expression of miR-181a, 135a, and 302c is mediated by DNA methylation in colon cancer. Shi et al. proved that dysregulation of these microRNAs promotes 5-FU resistance in microsatellite instable (MSI) CRC. Restoration of microRNAs expression attenuates PLAG1 expression and was shown to re-sensitize 5-FU resistant MSI CRC cell lines [78].

Another microRNA associated drug resistance is miR-149. A previous study showed that aberrant methylation is the main mechanism that is responsible for the silencing of miR-149 in CRC [79]. The expression of miR-149 is downregulated in 5-FU resistant cells as compared with their parental cells. Re-expression of this microRNA was able to enhance the 5-FU sensitivity of CRC cells by suppressing FOXM1 gene [80]. In addition, another recent study demonstrated that the upregulation of miR-149 expression together with the DNA de-methylation (5-aza-dc) therapy could positively elevate the chemosensitivity of CRC [65]. Similarly, the co-administration of dichloroacetate (DCA) and overexpression of miR-149 in CRC was shown to not only improve 5-Fu apoptosis, but also to help in minimizing glucose metabolism [81].

Other downregulated microRNAs, such as miR-200 [82], miR-17-5p [83], miR-124, miR-506 [84], miR-143 [85], and miR-340 [86] were associated with chemoresistance of multi-drugs in CRCs. The downregulation of these microRNAs was correlated with DNA methylation [87,88,89,90]. Considered together, these data suggest that epigenetic silencing of microRNAs has strong potential as a marker to predict chemotherapy response in CRC.

## 4. Conclusions

The methylation of a subset of microRNA genes could serve as a useful biomarker for the improvement of cancer detection and/or clinical outcome prediction. In addition, the restoration of epigenetically silenced tumor-suppressive microRNAs in cancer cells using the demethylating drugs could be a promising cancer treatment strategy. In the future, we envisage that further cancer epigenome and microRNA studies will lead to the discovery of a range of new biological markers and potential therapeutic targets.

## Data Availability

Not applicable.
